# Obstetric Section of New York Academy of Medicine

**Published:** 1880-06

**Authors:** 


### Reports of Societies.

### OBSTETRIC SECTION OF NEW YORK ACADEMY OF MEDICINE:

Dr. H. J. Garrigues read a paper which was a revised edition of his former paper on the subject, read September 8, 1877, and published in the "Proceedings Kings County Medical Society." The question was: "How long should a woman remain in bed after confinement?" It was desirable that practice, in this particular, should be as uniform as possible, and the author believes that the views entertained should not be so divergent as at the present time.

The chief representative of those who recommended that the time should be shortened as much as possible, was Dr. Wm. Goodell, of Philadelphia. At this point Dr. Garrigues referred to a case in which the woman was urged by her medical attendant to rise early, and she rose on the fourth day after delivery; on the fourteenth day she was induced to ride in a carriage, but it was nearly at the cost of her life. From that single illustration, however, he did not wish to draw any definite conclusions.

At the time Dr. Goodell's paper was read, 756 cases were reported, with a total mortality of only six; and the chief reasons why its author recommended early rising after delivery were the following: 1. Labor, if it was a physiological process, should not be made to wear the livery of disease. 2. The upright position excites the uterus to contact and thereby lessens the amount and duration of the lochia. 3. Uterine diseases are not known among the nations whose women rise early after delivery; and 4. Experience has shown that convalescence is far more prompt and sure than when the woman is kept in bed for a prolonged period. To these points Dr. Garrigues directed the attention of the Section. He maintained that although parturition was a physiological process, it was one in which the transition from the normal to the pathological condition was extremely common; and that was especially true of women of modern times. Again, if the upright position favored the discharge of lochia and diminished its amount and lessened its duration, it must also be borne in mind that serious displacements were liable to be produced by placing the woman in that position before the changes incident to the postpartum state had gone on sufficiently to enable the tissues of the pelvis to resist properly superincumbent weight and pressure; and, therefore, by other means should the influence of the lochia be modified. While it be true that uterine disease did not apparently exist among the women of nations where early rising after delivery was commonly practised, there were two factors by which such a conclusion must be modified when applied to modern civilized women; first, not much was known of uterine disease in ancient nations, and, second, modern women, with all the ennervating influences of what is termed civilization, cannot resist disease as did the ancient or the modern uncivilized matrons.

With reference to the good results obtained by Dr. Goodell, he thought they were due to the general excellent care given to his patients, rather than to early rising; and besides he thought it impossible to judge of final results by those obtained in the average length of time which the woman remained in the retreat after delivery.

Dr. Garrigues then quoted from leading authorities in the three chief countries in Europe, all of whom recommended absolute rest in the horizontal position for one, two and even three or four weeks after parturition. In New York, also, most obstetricians favored the long period of retention in bed after delivery.

The author of the paper then directed attention to anatomical and physical facts relating to the measurements and weight of the uterus after parturition, as given by the different observers, Berner, Henchell, and supplemented by Kustner in the *Archiv fur Gynæcologie*, Funfzehnter Band, 1879. He also referred to Dr. Van de Warker's paper in the *American Journal of Obstetrics* for July, 1878, Dr. Frank P. Foster's paper on the "Topographical Anatomy of the Uterus and its Surroundings," in the same journal for January, 1880, and then spoke of the changes that occur in the uterine mucous membrane and the muscles and skin of the abdomen.

In the language of the author of the paper, "anatomy and physiology teach us that the puerperal uterus is large, heavy, flabby, anteverted and anteflected; that all the surrounding parts destined to support it are distended, soft and yielding; that its interior presents one large wound, bathed in a rich fluid from disintegrated tissue elements; that the placental site is pervaded by large venous sinuses, filled with recently-formed blood clots; that at least the vaginal orifice and other parts of the obstetric canal present open wounds; that the process of transformation, absorption and regeneration required at least two months; and that the retrogression is most active during the second week."

It was not necessary that the woman should lie upon her back after the first twenty-four hours, but her position might be changed to that upon either side. The liability to hemorrhage, displacement of thrombi causing sudden death, and the occurrence of septicæmia, was regarded as sufficient reason for insisting upon rest in the horizontal position for several days at least after delivery. The vagina should be kept clean with disinfectant injections It was with reference to rest after delivery in normal childbirth that it was desirable to reach a unanimity of opinion. Upon that point Dr. Garrigues had reached the conclusion, from the combined teachings of experience and physiology, that the woman should be kept lying quietly in bed, alternately upon the back and side, until the uterus has contracted sufficiently to be behind the symphysis, and for two months she should avoid any great exertion.

Dr. Isaac E. Taylor remarked that the views held by Dr. Goodell were substantially those entertained by Hamilton and White, and published several years ago. The important point, however, was with reference to the management of the woman after normal natural labor, and he did not agree with Dr. Goodell, because he believed that we must be guided by the nature of the case under observation: what was the woman's physiological condition? what was the condition of the uterus as regards its length, weight and position? etc.

Dr. Taylor then referred to a case in which the uterus returned to the pelvic cavity within five days after delivery, and the woman made a rapid and good recovery; but not every case progressed so favorably as that one. He kept the woman in bed until the uterus had returned to the pelvic cavity, whether it required one or four weeks. So far as rest after delivery was concerned, we must judge by the constitution of the woman. Rising within two or three days and sitting on a vessel would, doubtless, facilitate removal of clots and also the lochia; but if the woman suffered formerly a good deal from the discharge, etc., he kept her in bed three or four weeks. There could be no line drawn or rule laid down which could be made applicable to every case.

Dr. Sell remarked that if the woman was properly bandaged and a pad was worn, getting up and down would not displace the uterus, and would favor discharge of the lochia. But the management of the puerperal period must be left to the judgment of the physician at the bedside of the woman. So long as the uterus could be felt above the brim of the pelvis, women in Vienna were not allowed to leave their beds.

Dr. F. V. White spoke of the efficacy of washing out the vagina with Labarraque's solution, in disinfecting the lochia and preventing the development of puerperal fever.

Dr. S. T. Hubbard remarked that he had found a great difference among women with reference to the time after delivery at which they could get up without injury. His rule had been to keep them in the recumbent posture, if possible, nine or ten days, and prevent them from walking for two weeks. He thought the time must be regulated by the attending physician, without reference to any rule.

Dr. Tausky fully agreed with Dr. Garrigues, and also believed that an important factor in preventing the development of puerperal fever was maintaining the recumbent posture after delivery for a week or more. He referred to a case in which the woman, feeling perfectly well on the fifth day after a normal labor, arose, and puerperal fever immediately followed. Some women might get up on the first day after delivery and no harm follow; and so it occasionally occurred that a person fell from a third-story window and received no serious injury, but he regarded such as exceptional cases, and thought that no woman should rise before the eighth or ninth day after a normal labor. He also approved of injections of the cavity of the body of the uterus, as recommended by Hegar, whenever the internal os was patulous.

Dr. O'Sullivan practiced retention in bed after delivery for nine or ten days, and as much longer as possible. He did not approve of vaginal or uterine injection, believing that if proper contraction was secured the uterus would be properly emptied.

Dr. J. N. Merrill endeavored to keep the lying in woman in bed from nine days to three weeks. He believed that uterine disease was very prevalent among the poor classes.

The chairman remarked that we need not go to Rome to study Roman women, for they were here; and he then referred to his experience among Italian women in the city of New York, which had been that early getting up after delivery frequently destroyed the life of the woman, and was a most prolific source of all kind of pelvic disease. He never allowed a woman to rise, if it could be prevented, before the ninth or ten day. He regarded cleanliness as godliness, but it was a virtue which most of the Italian discarded; and doubtless their habits in that respect contributed largely to the development of diseases among them.

Dr. Garrigues, in closing the discussion, remarked that he took it for granted that there were injuries more or less severe to the obstetric canal in every case of labor. The injury might be very slight, but it was sufficient to permit the absorption of septic material; hence the care that should be taken to keep the passage properly cleansed and the discharges properly disinfected.

The minimum time which he would keep the woman in bed was eight days, a period long enough to allow granulation to form for the repair of injury done to the tissues of the obstetric canal.

---

